# A systematic review of psychosocial interventions for children affected by armed conflict: mapping what works, for whom, and under what conditions

**DOI:** 10.1186/s40359-026-04726-9

**Published:** 2026-06-13

**Authors:** Atefeh Shamsi, Robabe Khalili

**Affiliations:** Nursing Care Research Center, Clinical Sciences Institute, Nursing Faculty, Baqiyatallah University of Medical Sciences, Tehran, Iran

**Keywords:** Armed conflict, Children, Adolescents, Psychosocial intervention, Mental health services

## Abstract

**Background:**

Armed conflict exposes children to prolonged and multifaceted adversity, including violence, displacement, bereavement, family disruption, poverty, insecurity, and disrupted schooling. Although research on psychosocial interventions for conflict-affected children has grown, the evidence remains heterogeneous, and causal conclusions are often difficult to distinguish from feasibility, contextual, or implementation findings.

**Objective:**

This systematic interpretive mapping review aimed to synthesize evidence on psychosocial interventions for children affected by armed conflict by mapping intervention types, delivery platforms, mechanism-related intervention content, family and caregiving context, and implementation conditions, while explicitly separating levels of evidentiary inference.

**Methods:**

A systematic search of five major databases identified 7,729 unique records, of which 50 evidence sources met the final inclusion criteria. Evidence sources included randomized controlled trials, cluster randomized trials, crossover randomized trials, quasi-experimental studies, pilot and usability studies, observational studies, reviews, conceptual papers, guidelines, operational guidance, and implementation sources. Data were extracted and synthesized using a convergent segregated mixed-methods approach. Evidence sources were classified into 17 effectiveness studies, 2 non-comparative intervention studies, and 31 contextual or implementation evidence sources to separate cautious causal inference from feasibility, contextual, policy, and implementation interpretation. Methodological quality was assessed using Joanna Briggs Institute tools where applicable, and certainty of evidence was assessed separately from study-level methodological quality.

**Results:**

Effectiveness evidence was concentrated in school-based, community-based, trauma-focused, group-based, family-focused, and emerging digital interventions, often delivered by non-specialist providers in resource-constrained or humanitarian settings. Controlled studies suggested modest and heterogeneous improvements in post-traumatic stress, depression, anxiety, behavioral difficulties, and psychosocial functioning. Family-focused and caregiver-relevant interventions appeared promising, but the number of rigorous trials was limited. Trauma-focused and skills-based interventions showed potential benefit when delivered with adequate structure, supervision, cultural adaptation, and continuity. Digital interventions remained at an early stage of evaluation. Contextual and implementation evidence highlighted the importance of caregiver functioning, ongoing daily stressors, displacement, safety, school continuity, supervision, service access, and delivery platform stability, but these factors were rarely tested as formal mechanisms or moderators.

**Conclusions:**

Psychosocial interventions for conflict-affected children show promise, but outcomes vary across intervention types, populations, delivery platforms, and conflict settings. Rather than identifying a single optimal intervention, this review suggests that intervention benefit should be understood within a broader ecological and implementation system. Future research should directly test mechanisms, moderators, developmental differences, implementation conditions, equity, digital access, and long-term outcomes to strengthen causal understanding and improve intervention design in humanitarian settings.

**Supplementary Information:**

The online version contains supplementary material available at 10.1186/s40359-026-04726-9.

## Introduction

Armed conflict profoundly reshapes the developmental ecology of childhood, disrupting not only individual psychological processes but also the relational, institutional, educational, and societal systems that support mental health. Exposure to violence, forced displacement, bereavement, family separation, material deprivation, and chronic insecurity is associated with elevated risks of post-traumatic stress, depression, anxiety, functional impairment, and behavioral dysregulation among children and adolescents [1–3]. These consequences should not be understood solely as responses to discrete traumatic events. Rather, they often develop within prolonged and changing conditions of instability, where daily routines, caregiving structures, school access, peer relationships, and community institutions are repeatedly disrupted. Childhood exposure to armed conflict therefore represents a complex and enduring public mental health challenge, rather than a time-limited clinical problem.

Global estimates indicate that large numbers of children live in conflict-affected or displacement settings, with many experiencing clinically meaningful psychological distress [1–3]. However, epidemiological patterns vary substantially across contexts, reflecting differences in type and intensity of exposure, phase of conflict, displacement trajectory, family resources, service access, and post-conflict or resettlement conditions. This variation is important for intervention research because conflict is not a static exposure. Children may be living during active violence, under occupation or siege, in temporary displacement, in protracted refugee situations, or in post-conflict reconstruction settings. These phases can shape both mental health needs and the feasibility of intervention delivery. Accumulating evidence indicates that child mental health outcomes are influenced not only by war-related traumatic events but also by ongoing daily stressors, including poverty, family strain, disrupted schooling, social isolation, insecurity, and limited access to services [4–6]. This has contributed to a shift from narrowly trauma-focused models toward broader ecological frameworks that conceptualize child mental health as embedded within interacting family, school, community, and service systems [5, 7].

Over the past two decades, a substantial body of research has examined psychosocial interventions for children affected by armed conflict and displacement. Prior systematic reviews and meta-analyses have reported modest but heterogeneous benefits across intervention types, including school-based group programs, trauma-focused therapies, family-oriented interventions, and community-based psychosocial supports [8–10]. Evidence syntheses have also emphasized that resilience, recovery, and intervention response are shaped by ecological and developmental conditions rather than by intervention content alone [11–13]. These reviews have highlighted persistent limitations in the evidence base, including variability in effect sizes, short follow-up periods, inconsistent outcome measurement, limited developmental stratification, and substantial heterogeneity in populations, delivery platforms, workforce arrangements, and implementation conditions. Accordingly, the present review does not claim to replace existing effectiveness reviews or meta-analyses. Instead, it seeks to complement them by mapping how different forms of evidence contribute to understanding the field.

Despite these advances, the literature remains conceptually and methodologically fragmented. Interventions are often described using overlapping or inconsistently defined labels, such as “community-based,” “school-based,” “psychosocial,” “trauma-focused,” or “resilience-building.” At the same time, key analytic dimensions such as intervention content, delivery platform, mechanism-related components, family context, and implementation conditions are not always clearly distinguished. This lack of conceptual separation can make it difficult to determine whether observed findings reflect intervention content, participant characteristics, delivery platform, facilitator training, family context, implementation continuity, or broader ecological constraints [5, 11–13]. Without such clarification, there is a risk of attributing observed outcomes to intervention content when they may also reflect differences in participant selection, baseline severity, attendance, facilitator training, supervision, family circumstances, setting stability, or service capacity.

In response to these challenges, the present review is framed as a systematic interpretive mapping review. Its purpose is not to provide a definitive causal answer to “what works, for whom, and under what conditions.” Rather, the review maps the structure of the evidence base, separates levels of inference, and identifies patterns that may inform future hypothesis testing. To preserve this distinction, the review consistently differentiates three evidence categories. First, effectiveness studies refer to randomized controlled trials and quasi-experimental studies that can contribute, with appropriate caution, to conclusions about intervention outcomes. Second, non-comparative intervention studies refer to single-arm, pre-post, pilot, feasibility, or usability studies that can inform acceptability, feasibility, implementation, and preliminary outcome signals, but cannot support causal conclusions. Third, contextual or implementation evidence sources refer to qualitative, observational, cross-sectional, measurement, policy-oriented, service-related, review, guideline, or conceptual sources that inform ecological interpretation, implementation conditions, and contextual understanding, but are not used as evidence of intervention effectiveness. This three-category distinction is applied throughout the review to avoid conflating causal inference with descriptive, contextual, or interpretive evidence.

Within this framework, the review organizes the literature along four analytically distinct but interacting dimensions: delivery platform, mechanism-related intervention content, family and caregiving context, and implementation layer. These dimensions are used as mapping categories rather than as confirmed causal mechanisms. Where primary studies directly test intervention effects, findings are reported as effectiveness evidence. Where studies describe feasibility, acceptability, attendance, implementation barriers, caregiver experiences, service conditions, or policy guidance, findings are treated as non-comparative or contextual evidence. This approach is consistent with humanitarian mental health guidance that emphasizes layered care, contextual adaptation, task-sharing, and coordination across health, education, family, and community systems [14, 15–17].

First, delivery platform refers to the institutional, organizational, or technological setting through which an intervention is delivered, such as schools, community centers, clinical services, home-based services, camps, or digital environments. Within this dimension, platform stability refers to the degree to which a setting can support predictable scheduling, regular attendance, continuity of facilitation, privacy, safety, and completion of intervention sessions. Platform stability is treated here as an implementation condition, not as a demonstrated causal mechanism. It may plausibly influence whether intervention components can be delivered with fidelity, but this relationship is considered an interpretive hypothesis unless directly tested in primary studies. This distinction responds to the concern that implementation conditions should not be described as mechanisms unless they have been formally tested.

Second, mechanism-related intervention content refers to the psychological processes or therapeutic components targeted by interventions, such as arousal regulation, emotional expression, cognitive restructuring, trauma narration, problem-solving, parenting support, or social skills. To differentiate levels of intervention intensity, the review uses the term mechanism depth to describe the extent to which an intervention engages these processes, ranging from low-intensity psychoeducation and coping skills to more intensive trauma-focused or family-based therapeutic work. This construct is used descriptively to organize intervention content. It is not used to infer that mechanisms were activated unless mediation, moderation, process, or component analyses were conducted in the original study. Accordingly, this review distinguishes intervention components from empirically demonstrated mechanisms of change.

Third, family and caregiving context is conceptualized as a multidimensional domain that includes caregiver mental health, parenting practices, family relationships, household stability, exposure to ongoing stressors, and relational climate. Ecological and observational evidence indicates that caregiver functioning, family relationships, and community context are closely related to child adjustment in conflict-affected settings [5, 6, 18, 19]. To avoid treating family functioning as a single undifferentiated construct, this review distinguishes four possible roles: family context as an intervention target, such as parenting or family-based programs,as a potential moderator of intervention response, such as caregiver distress or household instability; as an implementation condition, such as caregiver support for attendance and engagement; and as an outcome domain, such as changes in parent–child relationships or family functioning. This distinction is necessary because most included studies were not designed to formally test mediation or moderation pathways. Family and caregiving factors are therefore interpreted as important ecological and implementation dimensions, not as confirmed causal mechanisms unless directly tested.

Fourth, the implementation layer refers to the human, organizational, and supervisory resources required to deliver psychosocial interventions. This includes facilitator background, task-shifting arrangements, training, supervision, referral pathways, fidelity monitoring, and coordination with schools, community organizations, or health services. Non-specialist providers, teachers, lay workers, health workers, and nurses are considered within this broader implementation layer. Nursing roles are not positioned as a central theoretical axis of the review, because the available evidence does not support nursing as a distinct intervention category. Rather, nursing-related contributions are interpreted cautiously as part of screening, referral, coordination, psychoeducation, supportive care, and continuity of services where such roles are reported. This revision directly addresses the reviewer concern that nursing roles had previously been overstated.

In addition to clinical and implementation dimensions, psychosocial recovery in conflict-affected populations may involve broader social and intergroup processes. Armed conflict can reshape how children, families, and communities perceive former adversaries, moral responsibility, collective suffering, injustice, and possibilities for reconciliation. Emerging social-psychological research suggests that war-related memories, meta-humanization, empathy, perceived injustice, and solidarity-based action may influence intergroup attitudes and post-conflict social functioning [20–22]. These studies are relevant because they indicate that recovery after armed conflict may extend beyond individual symptom reduction to include social cognition, group relations, and reconciliation-related outcomes. However, because such processes were rarely measured in the intervention studies included in this review, they are incorporated as part of the broader conceptual context rather than treated as empirically established intervention mechanisms. This addition integrates the reviewer’s recommendation to consider intergroup relations theory while preserving clear evidentiary boundaries.

Taken together, these considerations highlight the need for a synthesis approach that distinguishes between evidence of intervention effects, preliminary intervention signals, and contextual or implementation insights. Accordingly, this review does not make prescriptive claims that a given intervention type is optimal for a given population or setting. Instead, it maps how intervention content, delivery platform, caregiving context, and implementation layer are represented across the literature, and it identifies where effectiveness evidence is stronger, limited, preliminary, or absent. This approach allows the review to generate cautious, testable hypotheses about how clinical content, delivery stability, family systems, and service capacity may interact in conflict-affected settings, while avoiding causal claims that exceed the available evidence.

The specific objectives of this review are therefore threefold: (1) to categorize psychosocial interventions for children affected by armed conflict according to delivery platform, mechanism-related content, family and caregiving context, implementation layer, and target population; (2) to summarize reported outcomes while maintaining explicit separation between effectiveness studies, non-comparative intervention studies, and contextual or implementation evidence sources; and (3) to synthesize contextual and implementation findings to develop an interpretive framework that can guide future research, hypothesis testing, and more precise intervention evaluation. By reframing the contribution in this way, the review provides a structured and transparent map of a heterogeneous field, complements existing systematic reviews and meta-analyses, and clarifies what can and cannot be concluded from the current evidence base.

## Method

### Review design

This review was conducted as a systematic interpretive mapping review using a convergent segregated mixed-methods design. This design was selected because the literature on psychosocial interventions for children affected by armed conflict includes heterogeneous evidence sources that serve different evidentiary functions. Randomized controlled trials and quasi-experimental studies can contribute to cautious conclusions about intervention outcomes, whereas pilot, feasibility, usability, observational, qualitative, review, conceptual, service, guideline, and implementation sources provide descriptive, contextual, or implementation-relevant evidence [9, 10, 23–25].

The purpose of the review was therefore not to generate a pooled estimate of effectiveness or to make definitive causal claims, but to map the structure of the evidence base while preserving clear boundaries between effectiveness evidence, non-comparative intervention evidence, and contextual or implementation evidence. This analytic positioning was adopted to avoid conflating causal inference with ecological, conceptual, policy, or implementation interpretation. Accordingly, the review applied a three-category evidence framework throughout the Methods, Results, tables, figures, and Discussion: (1) effectiveness studies, (2) non-comparative intervention studies, and (3) contextual or implementation evidence sources.

The review was conducted and reported in accordance with the PRISMA 2020 statement, with adaptations appropriate for a mixed-methods interpretive mapping review [26]. Because the included evidence sources varied substantially in design, population, intervention type, outcome measurement, and implementation context, several methodological safeguards were used to reduce the risk of overinterpretation. These included dual independent screening, dual data extraction, predefined evidence-category rules, design-specific quality appraisal where applicable, separate assessment of certainty of evidence, and explicit restrictions on the types of claims that could be drawn from each evidence category.

### Protocol status and methodological amendments

A structured protocol was developed to guide the review process. However, the review was not prospectively registered before screening began. An internal protocol document was finalized on 15 June 2025, after pilot screening but before full data extraction and synthesis. The absence of prospective registration is acknowledged as a methodological limitation because it may increase the risk of analytic flexibility.

Several methodological refinements were introduced after initial familiarization with the literature. These refinements included (1) extension of the search window to June 2025, (2) adoption of a convergent segregated mixed-methods synthesis structure, (3) development of the three-category evidence classification system, and (4) organization of the mapping framework around delivery platform, mechanism-related intervention content, family and caregiving context, and implementation layer. These refinements were made in response to the observed heterogeneity of study designs, intervention labels, outcome reporting, and implementation contexts.

To ensure transparency, these refinements are treated as post hoc methodological amendments rather than as fully a priori decisions. They were used to improve interpretability and prevent inappropriate aggregation across incompatible forms of evidence. To mitigate the risk introduced by non-prospective registration, the review applied explicit classification rules before final synthesis, retained an audit trail of screening and extraction decisions, and separated effectiveness conclusions from non-comparative and contextual interpretation throughout the manuscript.

### Eligibility criteria

Eligibility criteria were defined using an expanded PICOS framework and were supplemented by classification according to evidence function. Sources were eligible if they addressed children or adolescents aged 0 to 18 years who had been exposed to armed conflict, political violence, war-related insecurity, forced displacement, refugee status, or internal displacement. Sources with mixed-age samples were included only when child or adolescent data were reported separately or when the pediatric population formed the clear focus of the source.

Because developmental needs differ substantially between younger children and older adolescents, age range was extracted where available and considered during interpretation. However, formal age-stratified synthesis was not possible because most primary studies reported outcomes across broad age groups. This limitation is acknowledged in the interpretation of findings.

Eligible psychosocial interventions included trauma-focused therapies, trauma-focused cognitive behavioral therapy, narrative exposure therapy, skills-based and resilience-oriented programs, parenting and family-based interventions, school-based psychosocial programs, community-based psychosocial supports, task-shifted or non-specialist delivered interventions, and digital or technology-assisted interventions [8–10, 13]. Pharmacological-only interventions were excluded.

For the effectiveness evidence category, eligible comparators included randomized or quasi-experimental control conditions, such as treatment-as-usual, waitlist control, no-intervention control, enhanced usual care, or active comparator. Single-arm, pre-post, feasibility, usability, pilot, and training studies without a comparator group were retained for mapping purposes, but were classified as non-comparative intervention studies and were not used to support causal conclusions about effectiveness.

Qualitative, cross-sectional, observational, epidemiological, measurement, service, policy-oriented, review, commentary, guideline, and perspective sources were retained only when they contributed relevant contextual, ecological, implementation, measurement, service, policy, or conceptual information. These sources were not used to infer intervention effectiveness.

Primary outcomes included symptoms of post-traumatic stress disorder, depression, anxiety, behavioral difficulties, emotional difficulties, and psychological distress. Secondary outcomes included functional impairment, social adjustment, school-related functioning, caregiver-related outcomes, feasibility, acceptability, attendance, engagement, fidelity, supervision, service use, and other implementation indicators.

Additional inclusion criteria were publication in English between January 2010 and June 2025 for the primary intervention evidence base. Earlier foundational sources were retained only when they were needed to support background, conceptual framing, established intervention models, or implementation guidance, such as narrative exposure therapy, humanitarian MHPSS guidance, and prior reviews [14, 15, 27, 28].

### Evidence classification

To preserve breadth while maintaining inferential clarity, all included evidence sources were assigned to one mutually exclusive evidence category before synthesis. The three categories were defined as follows.

Effectiveness studies included randomized controlled trials, crossover randomized controlled trials, cluster randomized trials, quasi-experimental studies, and mixed-methods trials with intervention outcome evaluation and a comparator or controlled analytic structure. These studies were used to summarize intervention outcomes, with caution according to design quality, risk of bias, precision, consistency, and relevance to the review question.

Non-comparative intervention studies included single-arm studies, pre-post designs, pilot studies, feasibility studies, usability studies, and training evaluations without a comparator group. These studies were used to describe feasibility, acceptability, engagement, implementation processes, usability, and preliminary outcome signals. They were not used to make causal claims about intervention effectiveness.

Contextual or implementation evidence sources included qualitative studies, cross-sectional studies, observational studies, epidemiological studies, measurement studies, service studies, systematic reviews, conceptual papers, policy-oriented sources, operational guidance, commentaries, and perspective papers. These sources were used to inform ecological interpretation, family and caregiving context, implementation barriers and facilitators, service conditions, measurement issues, and setting-level constraints. They were not used to draw conclusions about intervention effects.

Following final eligibility assessment and classification audit, 50 evidence sources were retained and classified consistently across the PRISMA diagram, Tables 1 and 2, figures, and narrative synthesis. Based on the revised tables and cleaned reference list, these comprised 17 effectiveness studies, 2 non-comparative intervention studies, and 31 contextual or implementation evidence sources. This classification was applied throughout the manuscript to address the editor’s request for consistent reporting of evidence categories.

**Table 1:** Methodological appraisal, evidence category, and inferential contribution

ID	Source	Source type	Evidence category	Main methodological or interpretive issue	Contribution to synthesis
1	Tol et al. [29]	Cluster randomized trial	Effectiveness	Cluster design, implementation variability	Contributes to cautious effectiveness conclusions
2	Jordans et al. [30]	Cluster randomized trial	Effectiveness	Context-specific school delivery	Contributes to cautious effectiveness conclusions
3	Tol et al. [31, 32]	Cluster randomized trial	Effectiveness	Moderator findings context-specific	Contributes to effectiveness and moderation interpretation
4	Tol et al., [33, 34]	Cluster randomized trial	Effectiveness	Baseline and implementation variability	Contributes to cautious effectiveness conclusions
5	Diab et al., [35]	Cluster quasi-experimental study	Effectiveness	Non-random allocation, family moderation	Contributes cautiously to outcome interpretation
6	Punamäki et al., [36]	Randomized controlled trial	Effectiveness	Attrition and contextual instability	Contributes to cautious family-intervention evidence
7	Kangaslampi et al., [37]	Quasi-experimental/process analysis	Effectiveness-related	Posttraumatic cognition analysis	Supports mechanism-related interpretation, not definitive mediation
8	Pfeiffer et al., [38]	Randomized controlled trial	Effectiveness	Blinding not feasible	Contributes to effectiveness conclusions
9	Peltonen and Kangaslampi, [39]	Randomized clinical trial	Effectiveness	Multiple-trauma population, follow-up limits	Contributes to trauma-focused intervention evidence
10	McMullen et al., [40]	Randomized controlled trial	Effectiveness	Context-specific TF-CBT delivery	Contributes to trauma-focused intervention evidence
11	O’Callaghan et al., [41]	Randomized controlled trial	Effectiveness	Specific population, sexually exploited war-affected girls	Contributes to trauma-focused intervention evidence
12	Betancourt et al., [18, 19]	Randomized controlled trial	Effectiveness	Reintegration and youth behavioral outcomes	Contributes to effectiveness conclusions
13	Bryant et al., [42]	Randomized controlled trial	Effectiveness	Short-term distress outcomes	Contributes to effectiveness conclusions
14	Miller-Graff and Cummings, [43]	Randomized controlled trial	Effectiveness	Family-based intervention, setting specificity	Contributes to family-focused intervention evidence
15	Weisz et al., [44]	Randomized crossover trial	Effectiveness	Digital delivery, short follow-up	Contributes cautiously to digital intervention evidence
16	Bolton et al., [45]	Randomized controlled trial	Effectiveness	Adolescent war/displacement context	Contributes to intervention evidence in war-affected youth
17	Panter-Brick et al., [46]	Randomized evaluation and mixed-methods study	Effectiveness and implementation	Intervention and contextual components intertwined	Contributes to outcome and implementation interpretation
18	Pfeiffer and Goldbeck, [47]	Pilot study	Non-comparative intervention	No definitive causal inference	Feasibility and preliminary signal only
19	Segal et al., [48]	Development and usability study	Non-comparative intervention	Small usability sample, no effectiveness test	Feasibility and usability evidence only
20	Panter-Brick et al., [6]	Prospective observational study	Contextual or implementation	Observational associations	Contextual interpretation of caregiver-child mental health
21	Betancourt et al., [18, 19]	Observational/contextual study	Contextual or implementation	Residual confounding	Contextual interpretation of community characteristics
22	Miller and Rasmussen, [4]	Conceptual and empirical framework	Contextual or implementation	Not an intervention evaluation	Daily stressors and ecological interpretation
23	Miller and Jordans, [5]	Conceptual review	Contextual or implementation	Not an intervention trial	Interpretation of determinants of child mental health in war
24	de Jong et al., [49]	Epidemiological/commentary source	Contextual or implementation	Post-conflict mental disorder context	Conceptual and epidemiological context only
25	Reed et al., [1]	Review	Contextual or implementation	Not an intervention trial	Refugee and displaced child risk/protective factors
26	Fazel et al., [2]	Systematic review	Contextual or implementation	Adult and refugee mental disorder prevalence	Background prevalence context
27	Charlson et al., [3]	Systematic review and meta-analysis	Contextual or implementation	Population-level prevalence estimates	Conflict-setting mental disorder context
28	Tyrer and Fazel, [8]	Systematic review	Contextual or implementation	Heterogeneous interventions	School and community intervention context
29	Brown et al., [9]	Systematic review	Contextual or implementation	Review-level evidence	Background synthesis of child/youth interventions
30	Purgato et al., [10]	Systematic review and IPD meta-analysis	Contextual or implementation	Review-level evidence	Prior effectiveness synthesis context
31	Tol et al., [11, 12]	Systematic review	Contextual or implementation	Review-level resilience evidence	Resilience and mental health context
32	Jordans et al., [13]	Systematic review	Contextual or implementation	Review-level evidence	Prior psychosocial and mental health care evidence
33	Tol et al., [31, 32]	Research-priority paper	Contextual or implementation	Not an outcome evaluation	Research-priority context
34	Tol et al., [11, 12]	WHO recommendation commentary	Contextual or implementation	Recommendation summary	Policy and clinical guidance context
35	Tol et al., [33, 34]	Multisectoral conceptual paper	Contextual or implementation	Not an intervention trial	Multisectoral interpretation
36	Dorsey et al., [50]	Evidence-base update	Contextual or implementation	Review-level evidence	Trauma treatment evidence context
37	Kira et al., [51]	Theoretical paper	Contextual or implementation	Theory only	Continuous traumatic stress context
38	Robjant and Fazel, [52]	Review	Contextual or implementation	Review-level evidence	Narrative exposure therapy context
39	Morina et al., [53]	Meta-analysis	Contextual or implementation	Review-level evidence	PTSD intervention evidence context
40	Hoppen and Morina, [54]	Meta-analysis	Contextual or implementation	Review-level evidence	PTSD treatment evidence in trauma-exposed youth
41	Jordans et al., [55]	Process evaluation	Contextual or implementation	Implementation-focused	Implementation and delivery context
42	Jordans et al., [56]	Review and implementation source	Contextual or implementation	Review-level and implementation evidence	Humanitarian mental health care context
43	Spiegel et al., [57]	Health systems paper	Contextual or implementation	Not child-specific intervention evidence	Humanitarian health system context
44	Ventevogel et al., [58]	Commentary	Contextual or implementation	Research-practice focus	Implementation and policy context
45	IASC, 2007 [14]	Guideline	Contextual or implementation	Guideline, not empirical study	MHPSS policy and implementation context
46	WHO, 2013	Guideline	Contextual or implementation	Guideline, not empirical study	Stress-related condition guidance
47	WHO, 2016 [15]	Guideline	Contextual or implementation	Guideline, not empirical study	Task-sharing and mhGAP context
48	UNHCR, 2013 [16]	Operational guidance	Contextual or implementation	Operational guidance	Refugee MHPSS implementation context
49	UNICEF, 2018 [17]	Operational guideline	Contextual or implementation	Operational guidance	Community-based child and family MHPSS context
50	Naslund et al., [59]	Narrative review	Contextual or implementation	Digital mental health context, not child-conflict trial	Digital implementation context

**Table 2:** Characteristics of included evidence sources and role in synthesis

ID	Source	Country/region	Population or focus	Design/source type	Evidence category	Role in synthesis	Intervention or topic	Platform/context	Main outcome or focus
1	Tol et al., [29]	Indonesia	Children affected by political violence	Cluster randomized trial	Effectiveness	Outcome evidence	School-based mental health intervention	School	PTSD, distress
2	Jordans et al., [30]	Nepal	Conflict-affected children	Cluster randomized trial	Effectiveness	Outcome evidence	Classroom-based psychosocial intervention	School	PTSD, depression, functioning
3	Tol et al., [31, 32]	Sri Lanka	War-affected children	Cluster randomized trial	Effectiveness	Outcome and moderator evidence	Preventive school-based intervention	School	Mental health outcomes and moderators
4	Tol et al., [33, 34]	Burundi	War-affected children	Cluster randomized trial	Effectiveness	Outcome evidence	School-based mental health intervention	School	Mental health symptoms
5	Diab et al., [35]	Palestine	War-affected children	Cluster quasi-experimental study	Effectiveness	Outcome and family-context evidence	Psychosocial resilience intervention	School/community	Resilience, distress, family moderation
6	Punamäki et al., [36]	Gaza	War-affected children	Randomized controlled trial	Effectiveness	Outcome evidence	Family/sibling/peer intervention	Family/community	Psychological distress and resources
7	Kangaslampi et al., [37]	Palestine	War-affected children	Quasi-experimental/process analysis	Effectiveness-related	Mechanism-related interpretation	Psychosocial group intervention	Group/community	Posttraumatic cognitions
8	Pfeiffer et al., [38]	Kenya	Young refugees	Randomized controlled trial	Effectiveness	Outcome evidence	Trauma-focused group intervention	Community	PTSD, depression
9	Peltonen and Kangaslampi, [39]	Finland	Refugee children/adolescents	Randomized clinical trial	Effectiveness	Outcome evidence	Narrative exposure therapy	Clinical/community	PTSD and trauma symptoms
10	McMullen et al., [40]	DR Congo	Former child soldiers and war-affected boys	Randomized controlled trial	Effectiveness	Outcome evidence	Group TF-CBT	Community	PTSD and psychosocial outcomes
11	O’Callaghan et al., [41]	DR Congo	Sexually exploited war-affected girls	Randomized controlled trial	Effectiveness	Outcome evidence	Trauma-focused CBT	Community	PTSD and mental health symptoms
12	Betancourt et al., [18, 19]	Sierra Leone	War-affected youth	Randomized controlled trial	Effectiveness	Outcome evidence	Behavioral intervention	Community	Behavior and psychosocial functioning
13	Bryant et al., [42]	Jordan	Young Syrian refugees	Randomized controlled trial	Effectiveness	Outcome evidence	Brief group behavioural intervention	Community	Psychological distress
14	Miller-Graff and Cummings, [43]	Gaza	Youth and families	Randomized controlled trial	Effectiveness	Family-intervention evidence	Family-based intervention program	Family/community	Youth and family outcomes
15	Weisz et al., [44]	Ukraine/displacement settings	Displaced children and adolescents	Randomized crossover trial	Effectiveness	Digital outcome evidence	Digital problem-solving intervention	Digital	Depression and anxiety symptoms
16	Bolton et al., [45]	Northern Uganda	Adolescent survivors of war and displacement	Randomized controlled trial	Effectiveness	Outcome evidence	Depression-focused intervention	Community	Depression symptoms
17	Panter-Brick et al., [46]	Afghanistan	Youth in conflict-affected setting	Randomized evaluation and mixed methods	Effectiveness and implementation	Outcome and implementation evidence	Psychosocial intervention	Community	Distress, insecurity, mental health
18	Pfeiffer and Goldbeck, [47]	Germany/refugee context	Unaccompanied young refugees	Pilot study	Non-comparative intervention	Feasibility/preliminary evidence	Trauma-focused group intervention	Community/clinical	PTSD symptoms and feasibility
19	Segal et al., [48]	Israel/global relevance	Pediatric mental health during armed conflict	Development and usability study	Non-comparative intervention	Usability evidence	Digital mental health platform	Digital	Usability and acceptability
20	Panter-Brick et al., [6]	Conflict/refugee settings	Caregivers and children	Prospective observational study	Contextual or implementation	Caregiver-child contextual evidence	Not applicable	Family/community	Caregiver-child mental health
21	Betancourt et al., [18, 19]	Sierra Leone	War-affected youth	Observational/contextual study	Contextual or implementation	Community-context evidence	Not applicable	Community	Community characteristics and mental health
22	Miller and Rasmussen, [4]	Conflict and post-conflict settings	Conflict-affected populations	Conceptual/empirical framework	Contextual or implementation	Daily-stressors framework	Not applicable	Ecological context	War exposure and daily stressors
23	Miller and Jordans, [5]	War-affected settings	Children in war-torn settings	Conceptual review	Contextual or implementation	Ecological interpretation	Not applicable	Family/community/system	Determinants of child mental health
24	de Jong et al., [49]	Post-conflict settings	Post-conflict populations	Epidemiological/commentary source	Contextual or implementation	Epidemiological context	Not applicable	Post-conflict settings	Common mental disorders
25	Reed et al., [1]	Displacement/refugee settings	Displaced and refugee children	Review	Contextual or implementation	Risk/protective factor context	Not applicable	Refugee/displacement context	Mental health risk and protection
26	Fazel et al., [2]	Refugee resettlement settings	Refugees	Systematic review	Contextual or implementation	Prevalence context	Not applicable	Resettlement context	Serious mental disorder prevalence
27	Charlson et al., [3]	Conflict settings	Conflict-affected populations	Systematic review/meta-analysis	Contextual or implementation	Prevalence context	Not applicable	Conflict settings	Mental disorder prevalence
28	Tyrer and Fazel, [8]	Refugee/asylum-seeking settings	Refugee and asylum-seeking children	Systematic review	Contextual or implementation	Prior intervention context	School/community interventions	School/community	Intervention evidence overview
29	Brown et al., [9]	Armed conflict settings	Children and youth affected by armed conflict	Systematic review	Contextual or implementation	Prior review context	Psychosocial interventions	Multiple platforms	Common practice elements
30	Purgato et al., [10]	Low-resource humanitarian settings	Children in humanitarian settings	Systematic review/IPD meta-analysis	Contextual or implementation	Prior effectiveness context	Focused psychosocial interventions	Multiple platforms	Intervention effects
31	Tol et al. [11, 12]	LMIC armed conflict settings	Children and adolescents	Systematic review	Contextual or implementation	Resilience context	Not applicable	Ecological context	Resilience and mental health
32	Jordans et al., [13]	War-affected settings	Children in war	Systematic review	Contextual or implementation	Background treatment evidence	Psychosocial and mental health care	Multiple platforms	Treatment approaches
33	Tol et al., [31, 32]	Humanitarian settings	MHPSS research field	Research-priority paper	Contextual or implementation	Research-priority context	Not applicable	Humanitarian settings	Research priorities
34	Tol et al., [11, 12]	Humanitarian settings	Acute stress, PTSD, bereavement	Recommendation commentary	Contextual or implementation	Policy and clinical guidance	WHO recommendations	Humanitarian settings	Stress-related conditions
35	Tol et al., [33, 34]	Political violence settings	Conflict-affected populations	Multisectoral conceptual paper	Contextual or implementation	Multisectoral context	Not applicable	Health, education, social systems	Political violence and mental health
36	Dorsey et al., [50]	General trauma-exposed youth	Children and adolescents	Evidence-base update	Contextual or implementation	Trauma-treatment context	Psychosocial treatments	Clinical/community	Evidence base for trauma treatments
37	Kira et al., [51]	War and conflict settings	Conflict-affected populations	Theoretical paper	Contextual or implementation	Continuous traumatic stress context	Not applicable	Conflict settings	Continuous traumatic stress
38	Robjant and Fazel, [52]	Refugee/trauma settings	Trauma-exposed populations	Review	Contextual or implementation	NET context	Narrative exposure therapy	Clinical/community	Review of NET evidence
39	Morina et al., [53]	Trauma-exposed youth	Children and adolescents with PTSD	Meta-analysis	Contextual or implementation	PTSD treatment context	PTSD interventions	Clinical/community	Comparative outcome evidence
40	Hoppen and Morina, [54]	Trauma-exposed youth	Children and adolescents	Meta-analysis	Contextual or implementation	PTSD evidence context	Psychological interventions	Clinical/community	Single versus multiple trauma
41	Jordans et al., [55]	Conflict-affected settings	Children and services	Process evaluation	Contextual or implementation	Implementation evidence	Mental health care package	Service/community	Implementation process
42	Jordans et al., [56]	Humanitarian settings	Mental health care systems	Review/implementation source	Contextual or implementation	System-level interpretation	Evidence-based mental health care	Humanitarian systems	Implementation considerations
43	Spiegel et al., [57]	Conflict settings	Conflict-affected populations	Health systems paper	Contextual or implementation	Health-system context	Not applicable	Humanitarian health systems	Health-care needs
44	Ventevogel et al., [58]	Humanitarian settings	Child mental health systems	Commentary	Contextual or implementation	Research-practice context	Not applicable	Humanitarian systems	Research-practice gaps
45	IASC [14]	Global humanitarian settings	MHPSS systems	Guideline	Contextual or implementation	Policy framework	MHPSS guidelines	Humanitarian settings	Layered MHPSS support
46	WHO, 2013	Global	Stress-related conditions	Guideline	Contextual or implementation	Clinical guidance	Stress-related condition management	Health/humanitarian systems	PTSD, acute stress, bereavement
47	WHO, 2016 [15]	Global	Non-specialist health settings	Guideline	Contextual or implementation	Task-sharing context	mhGAP	Health systems	Mental health service delivery
48	UNHCR, 2013 [16]	Refugee operations	Refugee populations	Operational guidance	Contextual or implementation	Refugee MHPSS implementation	MHPSS programming	Refugee operations	Service implementation
49	UNICEF, 2018 [17]	Humanitarian settings	Children and families	Operational guideline	Contextual or implementation	Child and family MHPSS framework	Community-based MHPSS	Community/humanitarian systems	Three-tiered support
50	Naslund et al., [59]	LMICs/global	Digital mental health systems	Narrative review	Contextual or implementation	Digital implementation context	Digital mental health	Digital/global health	Access, scalability, implementation

### Search strategy and information sources

A comprehensive search was conducted on 1 July 2025 across PubMed/MEDLINE, PsycINFO, Scopus, Web of Science, and ProQuest Dissertations & Theses. The search strategy combined controlled vocabulary and free-text terms across three domains: conflict exposure, population, and intervention. Conflict-related terms included “armed conflict,” “war,” “political violence,” “refugee,” “internally displaced,” and “forced displacement.” Population terms included “child,” “children,” “adolescent,” “youth,” and “young people.” Intervention terms included “psychosocial,” “mental health support,” “psychological intervention,” “therapy,” “trauma-focused,” “school-based,” “community-based,” “parenting,” and “family intervention.”

The search strategy was informed by prior systematic reviews and methodological literature in humanitarian mental health and psychosocial support research [9–13]. Supplementary methods included forward and backward citation tracking of included studies and relevant reviews. Targeted searches of major humanitarian and global health sources, including UNICEF, WHO, UNHCR, and IASC materials, were conducted to identify implementation-relevant literature [14,15–17].

Peer-reviewed empirical intervention studies were used for effectiveness and non-comparative intervention synthesis. Reviews, guidelines, operational guidance, and conceptual sources were retained only as contextual or implementation evidence sources and were not counted as effectiveness studies (Table 3).

**Table 3:**
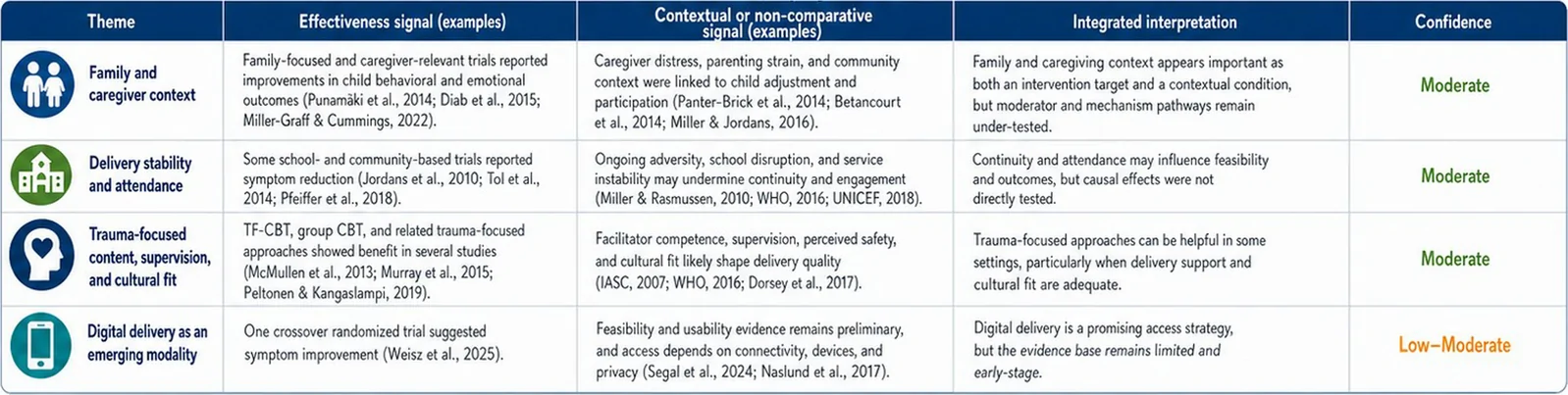
Joint display matrix (Miller and Jordans, [5] , Panter-Brick et al., [6], WHO, 2016 [15], UNICEF, 2018 [17], Betancourt et al., [18], Jordans et., [30], Tol et al., [33], Diab et al., [35], Punamäki et al., [28], Pfeiffer et al., [38], Peltonen and Kangaslampi, [39], McMullen et al., [40], Miller-Graff and Cummings, [43], Weisz et al., [44], Segal et al., [48], Dorsey et al., [50], Naslund et al., [59], Murray et al., [62]

Confidence reflects certainly in the integrated interpretation based on quantity, quality, consistency, and coherence of evidence across this map.

### Study selection

Following deduplication, 7,729 unique records were screened in two stages: title and abstract screening, followed by full-text review. Each stage was conducted independently by two reviewers. Disagreements were resolved through discussion and consensus, with third-party adjudication used when agreement could not be reached. Inter-rater reliability was substantial, with Cohen’s κ = 0.78 for title and abstract screening and κ = 0.84 for full-text review.

During final verification, study counts and source categories were audited to ensure consistency across the PRISMA diagram, Results section, Tables 1 and 2, figures, and narrative synthesis. This audit was conducted because earlier versions of the manuscript contained inconsistencies in the number of sources assigned to effectiveness synthesis, non-comparative intervention evidence, and contextual or implementation synthesis.

The revised classification uses the same totals throughout the manuscript: 50 included evidence sources, comprising 17 effectiveness studies, 2 non-comparative intervention studies, and 31 contextual or implementation evidence sources. Only the 17 effectiveness studies were used to support cautious conclusions about intervention outcomes. The 2 non-comparative intervention studies were used only to describe feasibility, acceptability, usability, implementation processes, and preliminary signals. The 31 contextual or implementation evidence sources were used only to inform ecological interpretation, implementation mapping, measurement issues, service context, policy context, or conceptual framing.

### Data extraction

Data extraction was conducted using a standardized and piloted extraction form. Extracted information included author, year, country or region, conflict or displacement context, participant characteristics, age range, sample size, study design or source type, evidence category, intervention type, delivery platform, facilitator type, intervention duration, comparator condition where applicable, outcomes, measurement tools, follow-up period, reported effect estimates where available, statistical significance, confidence intervals where available, implementation indicators, attendance, fidelity, supervision, acceptability, service factors, and contextual factors.

The extraction form also captured variables relevant to the interpretive mapping framework, including delivery platform, platform stability indicators, mechanism-related intervention content, mechanism depth, family and caregiving context, and implementation layer. These variables were used for structured mapping and hypothesis generation, not for causal attribution unless the original primary study directly tested the relevant pathway.

Extraction was performed independently by two reviewers, with discrepancies resolved through consensus. Where information was incomplete or ambiguously reported, the evidence source was retained only for the level of inference that the available data could support. For example, a pilot or usability study could contribute to feasibility mapping but not to conclusions about effectiveness; a guideline or conceptual paper could contribute to implementation interpretation but not to outcome synthesis.

### Quality appraisal and risk of bias

Methodological quality was assessed using Joanna Briggs Institute critical appraisal tools, selected because they can be applied across diverse empirical study designs [60]. Separate JBI tools were used for randomized controlled trials, quasi-experimental studies, qualitative studies, cross-sectional studies, observational studies, and other relevant empirical designs.

JBI appraisal was used only within study-design categories. Scores were not used to rank all sources globally and were not interpreted as equivalent indicators of causal evidentiary strength across designs. For example, a qualitative study could be judged methodologically rigorous for qualitative inquiry, but it was not treated as equivalent to a low-risk randomized trial for causal inference. Similarly, cross-sectional, measurement, service, commentary, guideline, and perspective sources could inform contextual or conceptual understanding but could not support conclusions about intervention effects.

Where numerical JBI summaries were retained, they were interpreted alongside domain-level judgments, including randomization, allocation concealment, baseline comparability, attrition, blinding where applicable, outcome measurement, confounding, reflexivity, sampling, and appropriateness of analysis. The primary purpose of quality appraisal was to inform interpretation within each evidence category, not to determine whether contextual or implementation sources could support intervention effectiveness claims.

Guidelines, manuals, reviews, and conceptual sources were not assigned causal risk-of-bias ratings. Instead, their role was restricted to contextual, implementation, policy, or conceptual interpretation.

### Synthesis of quantitative findings

Given substantial heterogeneity in interventions, populations, outcome measures, follow-up periods, and reporting formats, meta-analysis was not conducted. Instead, quantitative findings were summarized using a structured descriptive synthesis.

To avoid vote-counting and to preserve transparency, all available effect estimates were extracted where reported, including effect sizes, confidence intervals, p values, direction of effect, and follow-up timing. When effect sizes or confidence intervals were unavailable, the reported direction and statistical significance of findings were extracted, but such findings were interpreted cautiously. Outcomes were then summarized descriptively within intervention type and evidence category.

For effectiveness studies only, findings were categorized as: (1) statistically supported improvement, when between-group or controlled analyses showed benefit on a relevant outcome; (2) no clear evidence of change, when results were non-significant, underpowered, imprecise, or inconsistently reported; and (3) mixed or domain-specific findings, when effects differed by outcome, subgroup, follow-up period, or measurement domain. This categorization was used only as a descriptive summary layer and not as a substitute for meta-analysis. Non-significant findings were not interpreted as evidence of no effect, especially when studies were small, underpowered, or imprecise.

Non-comparative intervention studies were summarized separately. Pre-post improvements, feasibility indicators, usability findings, or engagement outcomes in these studies were described only as preliminary or implementation-relevant signals. They were not interpreted as evidence of effectiveness because such designs cannot separate intervention effects from regression to the mean, maturation, spontaneous recovery, contextual change, selection effects, or measurement effects.

Quantitative findings from contextual evidence sources, such as cross-sectional or epidemiological studies, were interpreted as descriptive or associational only. They were not combined with intervention outcome findings and were not used to support causal conclusions.

### Certainty of evidence

Certainty of evidence was evaluated at the body-of-evidence level using adapted principles from the GRADE framework [61]. Certainty ratings were assigned only to bodies of evidence addressing intervention outcomes and were based primarily on the 17 effectiveness studies. Contextual or implementation evidence sources were not assigned effectiveness certainty ratings. Non-comparative intervention studies were considered only as supplementary feasibility or preliminary evidence and did not increase certainty regarding intervention effectiveness.

Evidence was classified as high, moderate, low, or very low according to study design, risk of bias, consistency, precision, directness, and relevance to the review question. High certainty required consistent findings from multiple low-risk comparative studies. Moderate certainty reflected comparative evidence with some limitations, inconsistency, or imprecision. Low certainty reflected primarily non-randomized, heterogeneous, or methodologically limited comparative evidence. Very low certainty reflected evidence that was descriptive, preliminary, highly heterogeneous, or derived only from non-comparative or contextual sources.

This approach explicitly separates study-level methodological quality from body-of-evidence certainty. A source could receive a strong appraisal within its design while still contributing only limited certainty to effectiveness conclusions if it was non-comparative, observational, contextual, measurement-focused, service-oriented, guideline-based, or conceptual.

### Synthesis of qualitative, contextual, and implementation evidence

Qualitative, contextual, and implementation data were analyzed using thematic synthesis [23]. The synthesis focused on implementation barriers and facilitators, caregiver and family influences, setting characteristics, displacement-related constraints, safety and stability, cultural adaptation, workforce and supervision arrangements, service access, measurement issues, and broader ecological conditions.

Contextual and implementation evidence was synthesized separately from effectiveness studies. These findings were used to explain ecological and implementation conditions surrounding interventions, but not to establish whether interventions were effective. Cross-sectional findings were interpreted as associations only. Qualitative findings were interpreted as experiential, contextual, or implementation evidence. Measurement and service sources were used to inform understanding of assessment tools, service access, and implementation environments. Commentaries, guidelines, reviews, and perspective papers were used only for conceptual, policy, or implementation context and were not treated as empirical outcome evidence.

Findings from qualitative and contextual evidence were integrated with effectiveness and non-comparative intervention evidence using a joint display matrix [24, 25]. The matrix aligned intervention type, delivery platform, evidence category, target population, implementation conditions, and reported outcomes. This integration was used to identify recurring configurations of intervention, context, and outcome, while maintaining clear boundaries between empirical findings and interpretive inference. It was not used to infer causal mechanisms unless primary studies directly tested mediation, moderation, or mechanism activation.

### Management of mechanisms, moderators, and implementation hypotheses

To prevent overinterpretation, several analytic constraints were applied. Mechanisms were reported only when directly tested in primary studies through mediation analysis, process evaluation, component analysis, or other explicit mechanism-focused methods. Moderators were reported only when formally analyzed as interaction effects or subgroup effects.

Where studies suggested associations between attendance, caregiver functioning, delivery stability, supervision, cultural adaptation, service access, or contextual stressors and intervention outcomes, these were described as interpretive hypotheses generated by the mapping exercise, not as confirmed causal relationships. The review therefore avoids claiming that platform stability, family functioning, digital delivery, task-shifting, or implementation layer determines intervention effectiveness. Instead, these are treated as plausible explanatory dimensions that require direct testing in future research.

### Consistency checks before synthesis and reporting

Before final synthesis, all included evidence sources were checked against the revised evidence classification framework and against Tables 1 and 2. The final evidence-category totals were harmonized across the Methods, Results, PRISMA diagram, tables, figures, and Discussion. This step was undertaken to ensure that the manuscript consistently distinguishes between evidence sources that can contribute to cautious effectiveness conclusions, sources that provide only non-comparative intervention signals, and sources that provide contextual or implementation insight.

The final classification used throughout the manuscript is therefore: 17 effectiveness studies, 2 non-comparative intervention studies, and 31 contextual or implementation evidence sources. This classification preserves the breadth of the mapping review while preventing unsupported causal claims from non-comparative, observational, qualitative, cross-sectional, measurement, service, review, guideline, commentary, or perspective evidence.

## Results

### Study selection and structure of the evidence base

The study selection process yielded 7,729 unique records, of which 50 evidence sources met the final inclusion criteria following full-text screening. Because the revised evidence base included primary intervention studies, non-comparative intervention studies, reviews, conceptual papers, guidelines, operational guidance, and implementation sources, the term “evidence sources” is used where appropriate rather than “studies.” In response to concerns regarding heterogeneity and inappropriate aggregation of evidence, the included evidence sources were analytically partitioned into three mutually exclusive categories according to evidentiary function: effectiveness studies, non-comparative intervention studies, and contextual or implementation evidence sources.

Following final classification, 17 sources were categorized as effectiveness studies. These included randomized controlled trials, cluster randomized trials, crossover randomized trials, quasi-experimental studies, and mixed-methods studies with controlled intervention outcome evaluation [18, 19, 29–46]. Two sources were classified as non-comparative intervention studies, including pilot, feasibility, usability, or development studies without comparator groups [47, 48]. Thirty-one sources were classified as contextual or implementation evidence sources, including observational studies, reviews, conceptual papers, guidelines, operational guidance, implementation papers, and policy-relevant sources [1–6, 8–13], [14, 15–17]; [59].

This classification was applied consistently across the PRISMA diagram, Tables 1 and 2, figures, and narrative synthesis. Only the 17 effectiveness studies were used to support cautious conclusions regarding intervention outcomes. Non-comparative intervention studies were used to describe feasibility, acceptability, usability, engagement, implementation processes, and preliminary signals. Contextual or implementation evidence sources were used to inform ecological interpretation, service context, measurement issues, conceptual framing, policy context, and implementation constraints, but were not used to infer intervention effectiveness.

### Risk of bias and certainty of evidence

Methodological quality varied substantially across study designs and evidence categories. Among the effectiveness studies, common methodological concerns included unclear allocation concealment, baseline imbalance, attrition, short follow-up, implementation variability, and limited blinding [30–36, 42]. Performance bias was expected in most psychosocial intervention trials because participants, facilitators, teachers, lay workers, and service providers could rarely be blinded to intervention allocation.

To address inconsistencies between methodological quality and evidentiary strength, JBI appraisal scores were interpreted within study-design categories only and were not used to rank all evidence sources globally. This distinction was necessary because contextual, review, conceptual, guideline, and implementation sources may be useful for ecological or policy interpretation, but they cannot support causal claims about intervention effects (Aromataris and Munn, 2020). For example, a review or guideline could provide important implementation context, but it was not treated as equivalent to a randomized trial for causal inference.

Certainty of evidence was evaluated separately from JBI appraisal and was assigned only to bodies of evidence addressing intervention outcomes. Effectiveness studies contributed to outcome certainty judgments, whereas non-comparative and contextual or implementation evidence sources did not increase certainty regarding intervention effectiveness. This approach is consistent with the need to separate causal inference from contextual, ecological, and implementation interpretation in heterogeneous humanitarian mental health evidence [9, 10, 61].

Overall, certainty of effectiveness evidence was limited by heterogeneity in intervention content, outcome measurement, delivery platform, follow-up duration, and risk-of-bias profiles. Several controlled studies provided evidence of potential benefit, but the evidence base did not support uniform conclusions across all intervention types, populations, or conflict-affected settings [10, 30, 33, 34].

### Characteristics of included evidence sources

The included evidence sources were geographically concentrated in regions affected by armed conflict, forced displacement, or humanitarian service delivery. Effectiveness studies were conducted across settings including Indonesia, Nepal, Sri Lanka, Burundi, Palestine, Gaza, the Democratic Republic of Congo, Sierra Leone, Jordan, Kenya, Uganda, Finland, Zambia, and Ukraine-related displacement contexts [18, 19, 29–35, 40–42, 44]. Contextual and implementation sources included broader regional, global, and humanitarian guidance relevant to conflict-affected and displaced populations [14, 15–17].

Participants in the primary intervention studies ranged from early childhood to late adolescence, with most studies including children and adolescents between approximately 5 and 18 years of age. However, developmental stratification was rarely implemented. Most studies reported outcomes across broad age bands, limiting the ability to draw age-specific conclusions about intervention effects, feasibility, or acceptability [10, 30].

Interventions were delivered across multiple platforms, including schools, community settings, clinics, homes, refugee or displacement contexts, and digital platforms. School-based and community-based delivery were the most common platforms [29–34, 38]. Many interventions used task-shifted delivery models involving lay counselors, teachers, paraprofessionals, community workers, or non-governmental organization staff, reflecting the limited availability of specialist mental health professionals in humanitarian settings [15, 18, 19, 30, 62].

Follow-up durations were generally short. Most effectiveness studies reported outcomes immediately after the intervention or within a few months, limiting conclusions regarding durability of effects and long-term functional recovery. Evidence on stepped-care models, continuity of care, and sustained outcomes remained limited.

### Effectiveness evidence

Across the 17 effectiveness studies, psychosocial interventions showed promising but heterogeneous evidence for improving mental health and psychosocial outcomes among children affected by armed conflict. Reported benefits most commonly involved post-traumatic stress symptoms, psychological distress, depression, anxiety, behavioral difficulties, emotional regulation, and broader psychosocial functioning [29–35, 38–44, 62].

However, the magnitude, consistency, and durability of effects varied substantially across intervention types, delivery platforms, populations, and conflict settings. Several studies reported statistically supported improvements, but between-group differences were not always consistent, and follow-up periods were often short [30, 33, 34, 36]. Therefore, the evidence suggests that psychosocial interventions may be beneficial in some settings, particularly where interventions are structured, supervised, culturally adapted, and delivered with sufficient continuity. The evidence does not establish that any single intervention model is uniformly effective across conflict-affected contexts.

### School-based interventions

School-based interventions were among the most frequently studied delivery approaches. Controlled and cluster-based studies reported reductions in psychological distress, post-traumatic stress symptoms, or related mental health outcomes among conflict-affected children and adolescents [29–35]. These findings suggest that schools may provide a feasible and scalable platform for reaching children in humanitarian and displacement settings.

However, the evidence was not uniformly positive. Between-group differences were inconsistent across studies, and in some cases, improvements were also observed in comparison conditions [30, 33, 34]. This variability suggests that school-based delivery may depend on contextual factors such as attendance, school stability, facilitator capacity, supervision, and continuity of implementation. Broader ecological and implementation evidence also indicates that insecurity, displacement, poverty, school disruption, and service instability can undermine attendance and engagement [4, 5, 14, 17].

**Fig. 1:**
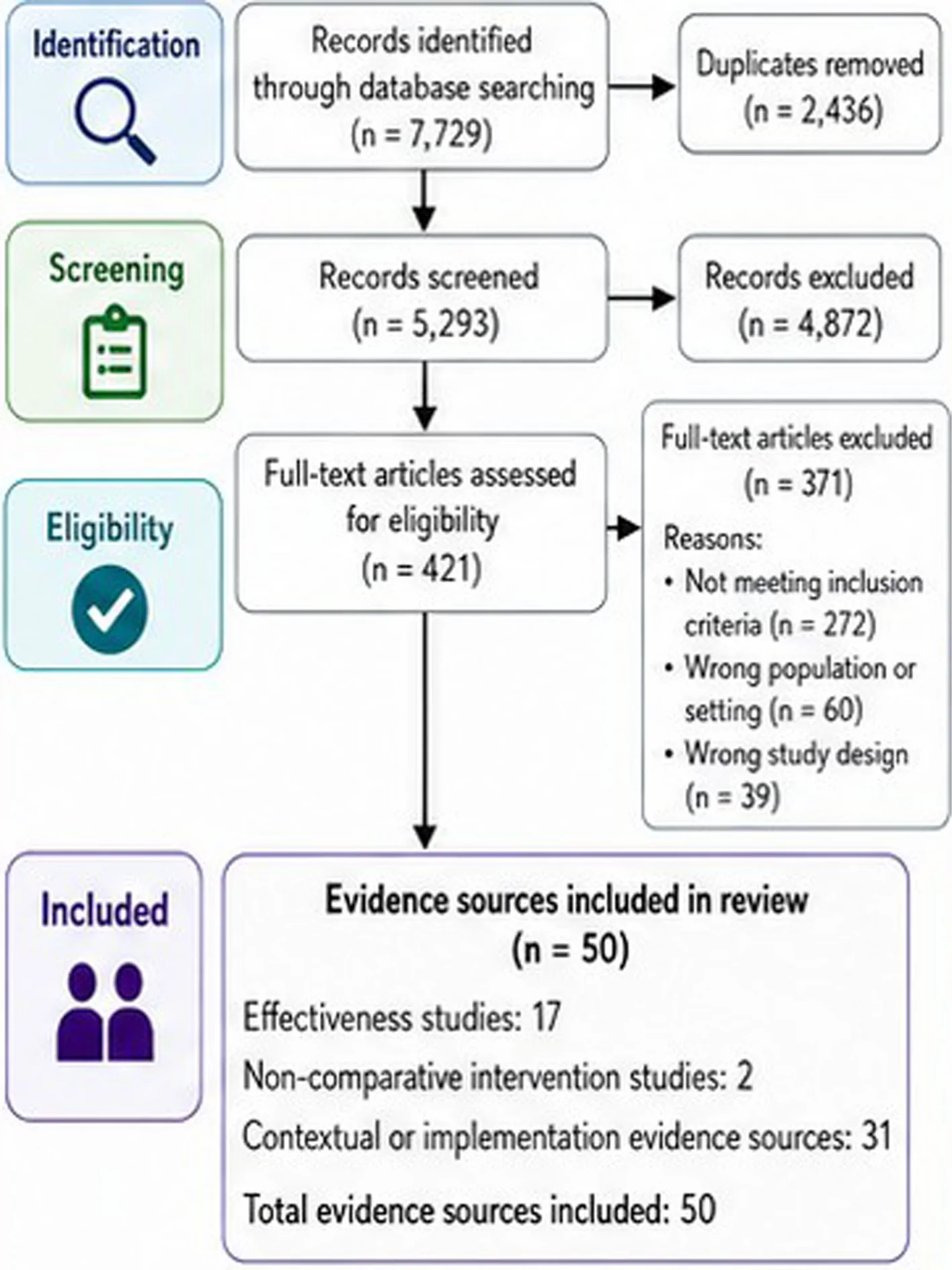
PRISMA-style flow diagram

**Fig. 2:**
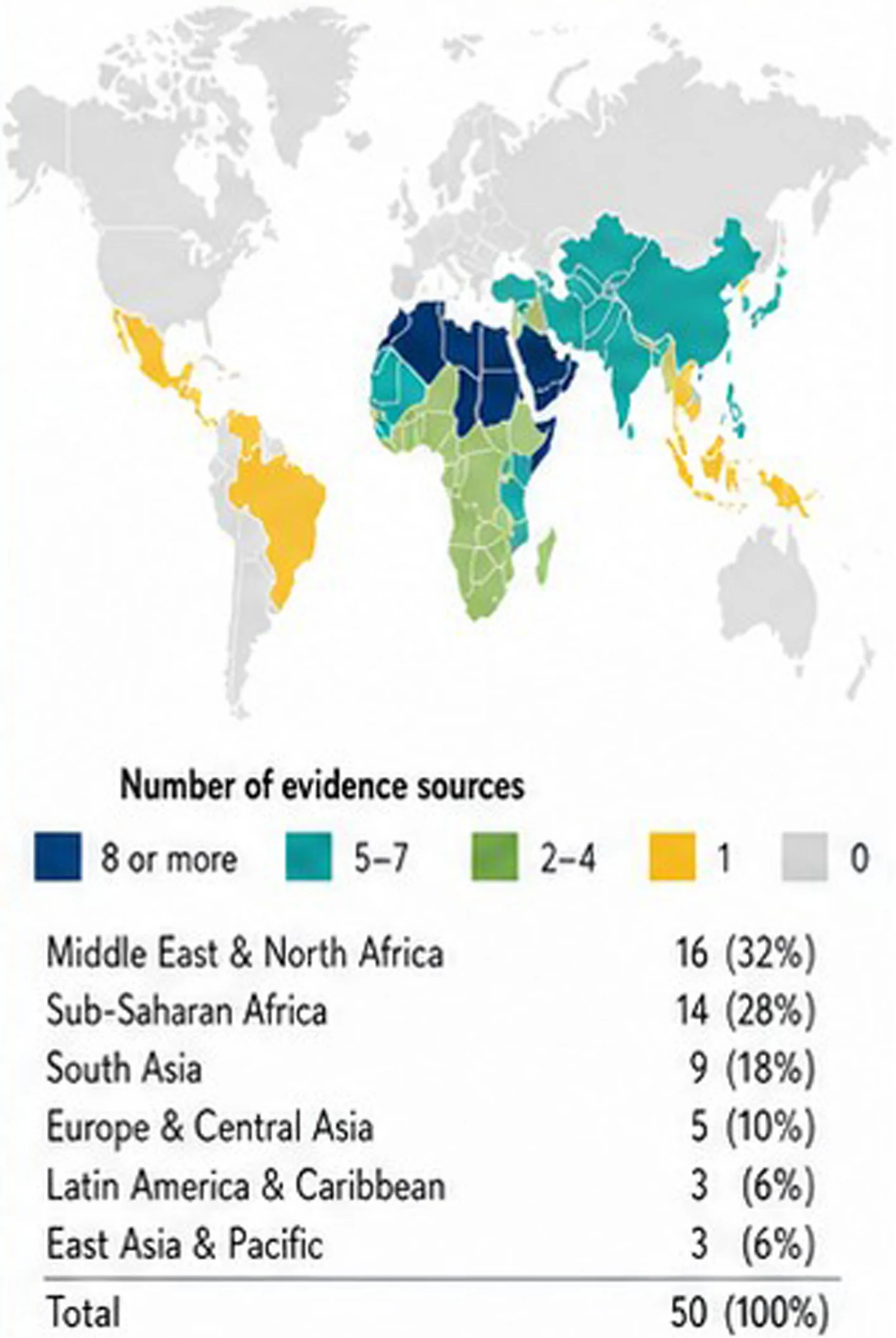
Geographic distribution of included evidence sources

**Fig. 3:**
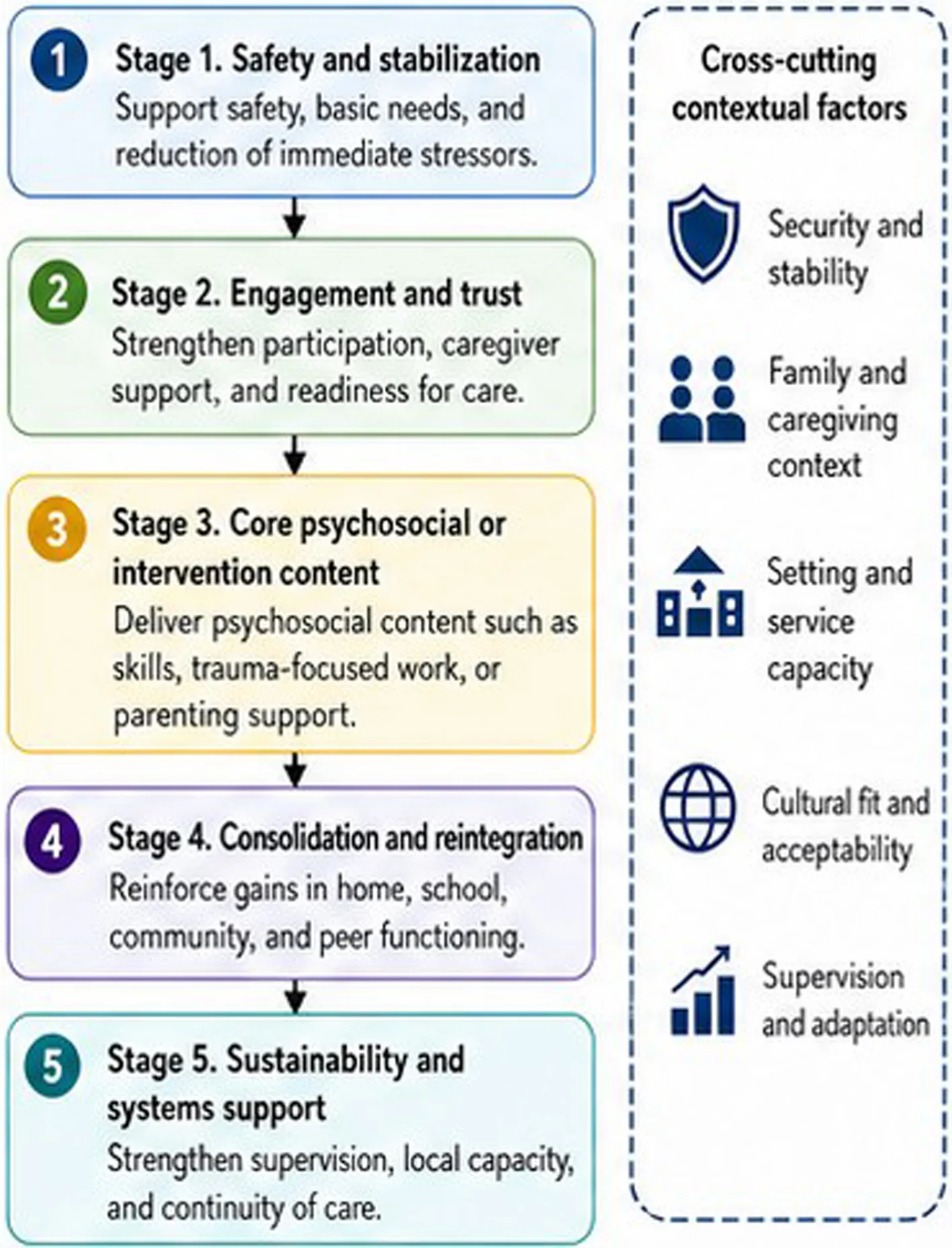
Interpretive mapping framework

**Fig. 4:**
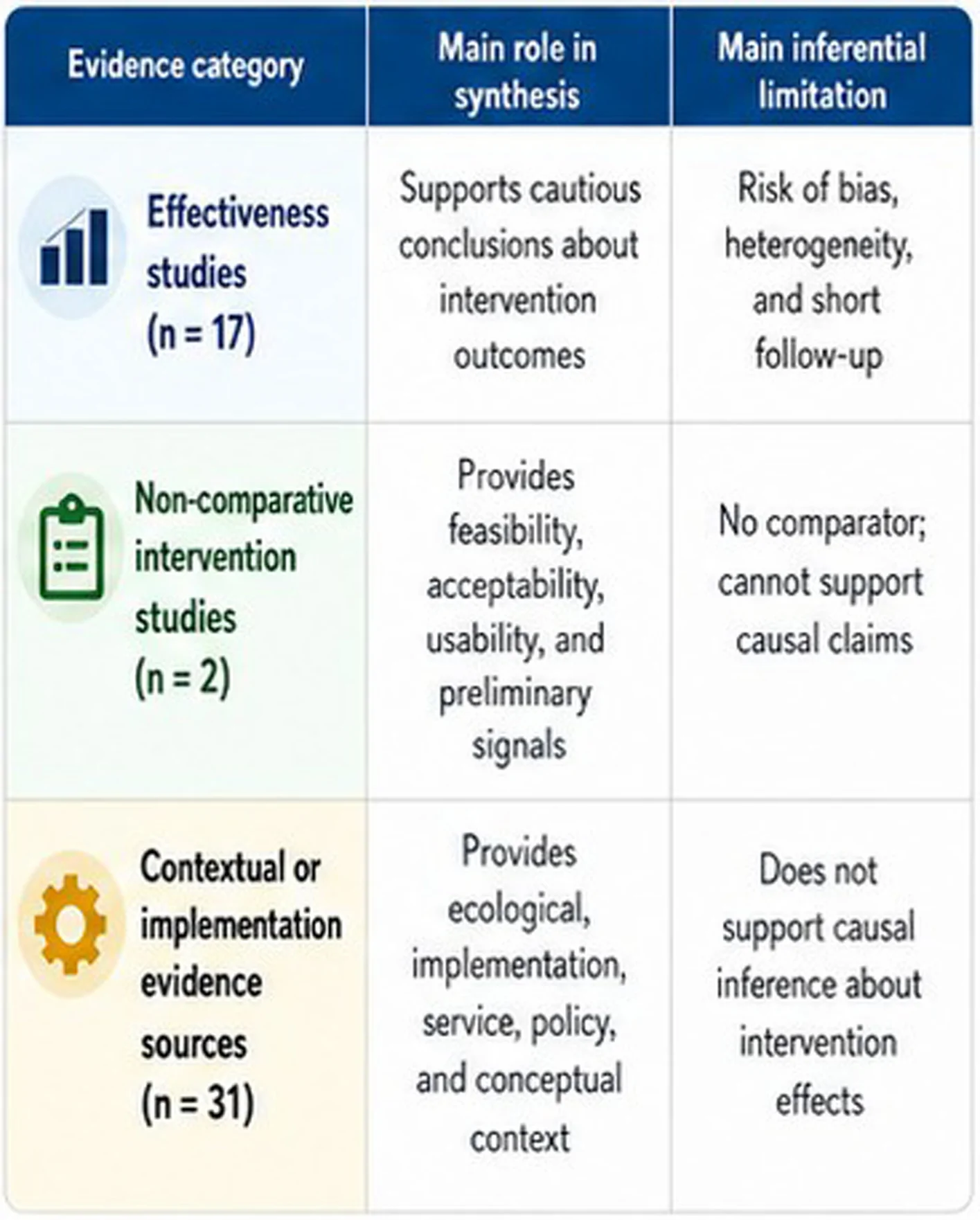
Evidence categorty and appraisal summary

Accordingly, school-based interventions are best interpreted as a potentially useful delivery platform rather than as a consistently effective intervention model. Their likely value depends on intervention content, implementation quality, attendance, supervision, and the stability of the educational environment (Figs 1, 2, 3, 4).

### Family and parenting interventions

Family and parenting interventions showed promising evidence, particularly for child behavioral and emotional outcomes. Randomized or controlled studies of family-focused or caregiver-relevant interventions reported improvements in child behavior, caregiver practices, family functioning, or child psychosocial outcomes [18, 19, 35, 36, 43]. These findings are consistent with ecological models emphasizing the role of caregiving environments and daily stressors in shaping child adjustment during and after armed conflict [4–6].

Nevertheless, the evidence base remained limited. Studies differed in intervention content, comparison condition, population characteristics, outcome measures, and follow-up timing. Effect estimates were not always precise, and the number of rigorous family-focused trials remained small. Therefore, family-based interventions should be described as a promising and theoretically well-supported area of intervention, not as a definitively superior approach.

Contextual evidence reinforced the relevance of caregiver distress, parenting strain, family disruption, household instability, and community context for child adjustment and intervention engagement [5, 6, 18, 19]. However, these sources did not establish causal pathways. Family and caregiving context should therefore be interpreted as both a potential intervention target and an important contextual condition that may shape participation and outcomes.

### Community-based and group interventions

Community-based and group interventions, including trauma-focused cognitive behavioral approaches, group-based psychosocial support, narrative exposure approaches, and skills-based models, showed evidence of benefit in several effectiveness studies [38–42, 62]. Reported improvements most often involved post-traumatic stress symptoms, emotional regulation, psychological distress, anxiety, depression, or broader psychosocial functioning.

However, outcomes varied across settings. The apparent benefit of community-based and group interventions appeared to depend on implementation conditions such as facilitator training, supervision, participant engagement, cultural adaptation, safety, and continuity of delivery [14, 15, 38, 62]. These conditions were frequently discussed in contextual or implementation sources, but they were rarely tested directly as moderators or mechanisms.

Therefore, community-based and group interventions should be interpreted as potentially beneficial when delivered under supportive conditions. The current evidence does not justify a general claim that these interventions are effective across all humanitarian contexts.

### Digital interventions

Digital interventions represented an emerging but still limited area of evidence. One randomized crossover trial suggested that a digital problem-solving intervention may improve depression or anxiety symptoms among children and adolescents displaced by war [44]. A development and usability study also suggested that digital platforms may be acceptable and potentially useful for pediatric mental health support during armed conflict, but this evidence did not test effectiveness [48].

Digital delivery may help address access barriers in settings where specialist providers, safe spaces, transport infrastructure, or continuity of services are limited. However, current evidence remains insufficient to support strong conclusions about effectiveness, equity, engagement, safety, durability, data privacy, cultural adaptation, or scalability. Digital interventions should therefore be described as a promising access strategy with an early-stage evidence base rather than as an established intervention modality [59].

### Non-comparative intervention evidence

The 2 non-comparative intervention studies provided information on feasibility, acceptability, usability, and preliminary implementation signals. These sources included a pilot trauma-focused group intervention and a digital development/usability study without comparator-based effectiveness evaluation [47, 48].

These studies reported preliminary signals related to feasibility, acceptability, symptom change, usability, or engagement. However, these findings were not interpreted as evidence of effectiveness because non-comparative designs cannot distinguish intervention effects from regression to the mean, maturation, spontaneous recovery, contextual change, selection effects, or measurement effects. Instead, these studies were used to identify potentially feasible delivery approaches, implementation challenges, acceptability considerations, and intervention models requiring more rigorous evaluation.

Non-comparative evidence was particularly useful for understanding emerging or under-tested modalities, including trauma-focused group delivery for refugees and digital mental health support. These findings require confirmation in controlled studies before stronger conclusions can be drawn.

### Contextual and implementation evidence

The 31 contextual or implementation evidence sources provided important insights into the ecological, service, and implementation conditions surrounding psychosocial interventions for children affected by armed conflict. These sources included observational studies, systematic reviews, meta-analyses, conceptual papers, implementation sources, policy guidance, operational guidance, and humanitarian mental health frameworks [1–6, 56], [14, 15–17].

Contextual and implementation evidence consistently highlighted the importance of caregiver distress, family disruption, displacement, insecurity, poverty, school instability, social support, cultural adaptation, task-sharing, supervision, service coordination, and access to care [4–6, 18, 19, 55, 56]. These findings are consistent with the daily stressors’ framework, which emphasizes that the mental health effects of armed conflict are shaped not only by exposure to violence but also by ongoing adversities, family stress, displacement, poverty, insecurity, and service limitations [4].

Within this perspective, psychosocial interventions operate within broader ecological systems. Their feasibility and apparent effects may be constrained or supported by contextual conditions such as safety, stability, caregiver functioning, social support, school continuity, facilitator capacity, supervision, and service access. However, these conditions were interpreted as contextual and implementation signals only. They did not establish causal pathways or intervention mechanisms unless directly tested in primary intervention studies. Guidelines and operational sources contributed additional information on layered care, task-sharing, referral pathways, coordination, and child- and family-centered mental health and psychosocial support in humanitarian settings [14, 15–17]. These sources were not treated as empirical outcome evidence.

### Integrated thematic synthesis

The joint synthesis of effectiveness, non-comparative intervention, and contextual or implementation evidence identified four recurring themes.

First, family and caregiver context appeared important both as an intervention target and as a contextual condition. Effectiveness studies of family-focused or caregiver-relevant approaches reported improvements in child behavioral, emotional, or family-related outcomes [18, 19, 35, 36, 43]. Contextual evidence highlighted caregiver distress, family instability, community context, and daily stressors as influences on child adjustment and participation [4–6, 18, 19]. However, caregiver functioning was rarely tested as a formal moderator or mediator.

Second, delivery stability and attendance appeared central to feasibility and potential intervention benefit. School and community interventions sometimes produced symptom reductions [29, 30, 33, 34, 38]. At the same time, contextual evidence showed that insecurity, displacement, disrupted schooling, service instability, and limited workforce capacity could undermine attendance and continuity [4, 14, 15, 17]. These findings suggest that delivery stability may be important, but its causal role remains under-tested.

Third, trauma-focused content, supervision, and cultural fit emerged as a recurrent configuration across several intervention types. Trauma-focused cognitive behavioral approaches and narrative exposure therapy showed benefit in some studies [38–41, 62]. However, the evidence does not establish that trauma processing alone explains outcomes. Mechanism-related interpretations remain tentative unless mediation, moderation, or process pathways were directly tested.

Fourth, digital delivery emerged as a promising but early-stage modality. One controlled trial suggested potential benefit [44], while usability evidence indicated possible reach and engagement advantages but remained preliminary [48]. Questions remain about equity, safety, engagement, data privacy, cultural adaptation, and long-term impact [59].

These themes should be understood as components of an interpretive mapping framework rather than as empirically validated causal pathways. The synthesis does not support definitive claims regarding “what works, for whom, and under what conditions.” Instead, it identifies patterns, constraints, and hypotheses that warrant further investigation.

### Nursing and non-specialist roles in implementation

Across the included evidence sources, nurses were mentioned in a limited number of cases and generally appeared in supportive roles, such as screening, referral, coordination, health education, psychoeducation, or linkage to services. The broader evidence base more consistently reflected reliance on non-specialist providers, including lay counselors, teachers, paraprofessionals, community workers, and non-governmental organization staff [18, 19, 30, 38, 62].

The available evidence does not support nursing actions as a distinct or central intervention domain, nor does it support claims that nursing involvement independently improves intervention effectiveness. Rather, nursing and related health-worker roles are best understood as part of a broader implementation layer that may support identification, referral, continuity of care, and coordination across services. This interpretation is consistent with global mental health approaches that emphasize task-sharing and system-level support in settings with limited specialist capacity [14, 15].

Accordingly, claims about nursing have been tempered. The Results support discussion of nursing as an implementation and systems-support role, but not as an independently established mechanism of intervention effectiveness.

### Overall synthesis of findings

Overall, the evidence base suggests that psychosocial interventions for children affected by armed conflict may produce modest improvements in mental health and psychosocial outcomes, particularly when delivered through structured, supervised, culturally responsive, and stable platforms. The strongest evidence comes from controlled studies of school-based, community-based, trauma-focused, group-based, family-focused, and emerging digital interventions [29–35, 38–44, 62].

However, the strength and consistency of effects are limited by heterogeneity, risk of bias, short follow-up periods, and variation in implementation conditions. Family-based interventions and task-shifted delivery models appear promising, while digital approaches remain emerging and underdeveloped [43, 44, 48, 59]. Contextual factors, including caregiver well-being, safety, displacement, service stability, school continuity, supervision, and cultural fit, appear important for understanding intervention feasibility and variability in outcomes, but their causal influence has not been definitively established [4–6, 18, 19].

The findings are therefore best interpreted as a structured map of the current evidence landscape rather than a definitive account of intervention effectiveness. The review identifies where evidence is strongest, where it remains preliminary, and where future studies should directly test mechanisms, moderators, implementation conditions, developmental differences, and long-term outcomes.

## Discussion

This review provides a structured interpretive mapping of psychosocial interventions for children affected by armed conflict, integrating effectiveness evidence, non-comparative intervention evidence, and contextual or implementation evidence sources. Rather than identifying a single superior intervention, the synthesis highlights recurring relationships between intervention type, delivery platform, family and caregiving context, and implementation conditions. The findings suggest that apparent intervention benefit is shaped not only by intervention content, but also by whether programmes can be delivered with sufficient continuity, supervision, cultural fit, and contextual support.

This framing responds to a long-standing challenge in humanitarian mental health research, where heterogeneous findings may reflect not only variation in intervention efficacy, but also differences in population characteristics, delivery systems, contextual adversity, workforce capacity, and implementation conditions [5, 9, 10, 13]. Accordingly, this review does not provide a definitive answer to “what works, for whom, and under what conditions.” Instead, it maps the evidence relevant to that question and identifies where the field has comparatively stronger evidence, where findings remain preliminary, and where future research should directly test mechanisms, moderators, and implementation conditions.

### Interpretation of the evidence base

The final synthesis included 50 evidence sources, classified into 17 effectiveness studies, 2 non-comparative intervention studies, and 31 contextual or implementation evidence sources. This three-category structure was central to the interpretation of findings. Effectiveness studies, including randomized controlled trials, cluster randomized trials, crossover randomized trials, quasi-experimental studies, and mixed-methods studies with controlled outcome evaluation, were used to support cautious conclusions about intervention outcomes. Non-comparative intervention studies were used to describe feasibility, acceptability, usability, engagement, and preliminary implementation signals. Contextual or implementation evidence sources were used to inform ecological interpretation, service conditions, measurement issues, policy context, conceptual framing, and implementation constraints.

This distinction is important because the review includes diverse forms of evidence that cannot support the same level of inference. A rigorous contextual, observational, review, guideline, or conceptual source may provide important insight into family distress, displacement, service access, policy context, or cultural fit, but it cannot establish intervention effectiveness. Similarly, a pilot or usability study may identify promising feasibility or engagement signals, but it cannot distinguish intervention effects from maturation, regression to the mean, spontaneous recovery, selection effects, or contextual change. By separating these evidence functions, the review avoids conflating causal inference with contextual interpretation.

Overall, the evidence suggests that psychosocial interventions may produce modest improvements in mental health and psychosocial outcomes for some children affected by armed conflict, especially when interventions are structured, supervised, culturally responsive, and delivered through stable platforms. However, the strength and consistency of this evidence are limited by heterogeneity in populations, intervention models, outcomes, follow-up periods, and risk-of-bias profiles. The evidence base therefore supports cautious, context-sensitive interpretation rather than broad prescriptive conclusions.

### Family and caregiving context

A central pattern emerging from the synthesis is the importance of family and caregiving context. Across effectiveness and contextual evidence, caregiver distress, parenting practices, family disruption, household instability, and community conditions were repeatedly relevant to child mental health, participation, and adjustment [5, 6, 18, 19]. These findings align with ecological and developmental models that conceptualize child adjustment in conflict settings as embedded within caregiving systems and daily stressors rather than determined solely by exposure to violence [4, 7].

Family-focused and caregiver-relevant trials provided promising evidence that interventions targeting caregiver behaviour, parenting practices, family relationships, or family support may improve child behavioural, emotional, or psychosocial outcomes [18, 19, 35, 36, 43]. However, the number of rigorous trials remains limited, and studies differ in intervention content, outcome measurement, comparison condition, follow-up period, and population context. Therefore, the findings do not establish that family-based interventions are definitively superior to other psychosocial approaches.

Within this review, family functioning is best interpreted as a theoretically important and empirically supported domain that may influence engagement and outcomes. It may operate as an intervention target, a contextual condition, a potential moderator, or a potential mediator, depending on study design and intervention model. However, these roles were rarely tested directly. Future studies should therefore specify whether caregiver and family variables are hypothesized to function as mechanisms, moderators, implementation conditions, or outcomes, and should measure them accordingly.

### Delivery stability, attendance, and implementation continuity

The review also identifies delivery stability and attendance as important dimensions shaping intervention feasibility and possible benefit. School- and community-based interventions dominated the effectiveness evidence base, but they were often delivered in environments characterized by insecurity, displacement, disrupted schooling, service instability, and variable access. Under such conditions, attendance variability and interruptions to programme delivery may alter the effective dose and continuity of psychosocial support [29–34, 38].

These findings are consistent with broader ecological literature emphasizing the importance of daily stressors and environmental instability for mental health in conflict-affected populations [4, 5]. However, the current evidence does not demonstrate that delivery conditions are more important than intervention content. Rather, it suggests that intervention content may be difficult to implement, receive, or sustain when safety, attendance, facilitator availability, supervision, and service continuity are unstable.

This distinction is important. The review does not support the claim that intervention content is secondary or unimportant. Instead, it identifies a sequencing and implementation problem: psychosocial intervention components may be less likely to achieve intended effects when children cannot attend sessions consistently, when facilitators lack supervision, or when insecurity disrupts continuity. Future trials should therefore measure attendance, fidelity, supervision intensity, facilitator competence, platform stability, and service continuity as core variables rather than as peripheral implementation details.

### Trauma-focused and skills-based intervention content

Trauma-focused and skills-based interventions, including trauma-focused cognitive behavioural therapy, group cognitive behavioural approaches, and narrative or exposure-related models, showed benefit in several effectiveness studies, particularly for post-traumatic stress symptoms and related distress outcomes [38–41, 62]. These findings are broadly consistent with child trauma literature indicating that trauma-focused approaches can reduce symptoms when delivered with adequate structure and support [50, 52, 53].

However, the mapped evidence also indicates that these approaches are not uniformly effective across settings. Their feasibility and apparent impact appear to depend on delivery conditions, including facilitator training, supervision, perceived safety, cultural acceptability, and continuity of care. In unstable settings characterized by ongoing threat, displacement, or disrupted services, trauma-processing components may be more difficult to deliver with fidelity or may require careful sequencing within layered care models [14, 15].

The included studies generally did not test mechanisms directly. Therefore, any interpretation that safety, supervision, or cultural fit influences trauma-focused intervention outcomes should be understood as an interpretive hypothesis generated by converging patterns across the evidence base, not as a confirmed causal pathway. Future research should directly examine mechanism activation, mediation, moderation, and context-by-intervention interactions, especially in high-risk and unstable environments.

### Digital and technology-assisted interventions

The evidence base for digital and technology-assisted interventions remains limited but evolving. One crossover randomized controlled trial suggested that a digital problem-solving intervention may improve symptoms among children and adolescents displaced by war [44]. A development and usability study also suggested that digital platforms may be acceptable and potentially useful for pediatric mental health support during armed conflict, but this evidence did not test effectiveness [48].

Digital delivery may expand access in settings where specialist providers, safe physical spaces, transport, or continuity of services are limited. However, digital interventions also introduce context-specific constraints, including device access, connectivity, privacy, data protection, caregiver involvement, literacy, language adaptation, and differential engagement. These factors may systematically shape who can access and benefit from digital support.

Consistent with global mental health literature on digital delivery [59], digital interventions should not be conceptualized as neutral delivery channels. They are context-dependent systems requiring dedicated evaluation of access, equity, safety, engagement, cultural fit, and durability. At present, digital interventions are best interpreted as a promising but preliminary modality, with limited confidence in the integrated interpretation.

### Social and intergroup dimensions of recovery

An important conceptual implication of this review is that psychosocial outcomes in war-affected children cannot be fully understood through symptom reduction alone. While the included intervention studies primarily focused on post-traumatic stress, depression, anxiety, distress, functioning, and behavioural outcomes, broader social and intergroup processes may also shape recovery in post-conflict settings.

Emerging research in social psychology highlights how war-related memories, group-based emotions, perceived injustice, empathy, meta-humanization, and solidarity-based action can influence intergroup attitudes and reconciliation-related processes [20–22]. These processes were not directly examined in most included intervention studies, and therefore they should not be treated as established mechanisms within this review.

Nevertheless, their relevance highlights a conceptual limitation of narrowly clinical frameworks. Psychosocial recovery may involve not only individual symptom change, but also social cognition, identity, belonging, trust, and intergroup orientation. Future research in conflict-affected child and adolescent populations may benefit from closer integration between psychosocial intervention research, developmental psychopathology, and intergroup relations theory.

### Nursing and non-specialist roles in implementation

The role of nursing and non-specialist providers requires careful interpretation. Nurses and allied health providers were identified only in limited and mostly supportive roles, such as screening, referral, health education, psychoeducation, coordination, and linkage to services. More commonly, interventions were delivered by lay counsellors, teachers, paraprofessionals, community workers, therapists, or non-governmental organization staff [18, 19, 30, 38, 62].

The available evidence does not support positioning nursing as a distinct or central theoretical axis of intervention effectiveness. Nor does it support claims that nursing involvement independently improves psychosocial outcomes. Rather, nursing and allied health roles are more appropriately understood as part of a broader implementation layer that may support identification, referral, continuity of care, and coordination within multi-tiered care systems [14, 15].

This interpretation aligns with task-sharing models in global mental health, which emphasize the role of non-specialist and community-based providers in extending access to mental health and psychosocial support in low-resource and humanitarian settings [15, 63]. Accordingly, the review supports discussion of nursing and non-specialist providers as implementation supports, but not as independently established mechanisms of intervention effectiveness. Methodological and reporting guidance for systematic reviews further supports transparent registration, protocol reporting, peer review of search strategies, systematic review conduct, and appraisal of qualitative evidence [64–68].

### Methodological and conceptual implications

This review contributes to the field by reframing heterogeneity as an object of analysis rather than only as a barrier to synthesis. The mapping approach highlights systematic relationships between intervention modality, delivery platform, family context, and implementation conditions. Rather than asking only whether interventions work on average, future research should examine how intervention components interact with delivery stability, caregiver functioning, facilitator capacity, cultural adaptation, and ongoing adversity. Low-intensity stress-management guidance and established trauma-focused CBT manuals may inform intervention content, but in this mapping review they should be treated as contextual practice resources rather than direct evidence of effectiveness in conflict settings [69, 70].

Several methodological priorities follow from this interpretation. Future process studies should directly test mediators and moderators, as prior school-based psychosocial intervention research has begun to do [71], and should address cultural adaptation challenges documented in humanitarian counselling contexts [72]. Future trials should prospectively measure attendance, fidelity, supervision, facilitator competence, caregiver functioning, perceived safety, displacement status, service continuity, and platform stability. Studies should also test whether these variables operate as moderators, mediators, or implementation determinants. Longer follow-up periods are needed to determine whether symptom improvements are sustained and whether interventions influence functioning, school participation, family relationships, and social reintegration.

In addition, the field would benefit from greater developmental specificity. Many studies combined children and adolescents across broad age ranges, limiting conclusions about developmental timing, intervention fit, and age-specific response. Future studies should report age-stratified outcomes where possible and should consider whether younger children, early adolescents, and older adolescents require different intervention targets, delivery formats, or caregiver involvement.

### Practice and policy implications

From a practice and policy perspective, the review supports a cautious and context-sensitive approach to psychosocial intervention planning in conflict settings. Existing evidence indicates that several intervention types may produce symptom improvements, but the data do not support strong prescriptive claims regarding optimal intervention matching across contexts. These conclusions are consistent with broader refugee PTSD treatment literature [73], evidence-practice gap discussions in humanitarian child mental health [74], and global mental health task-sharing literature emphasizing trained, supervised non-specialist delivery [75].

Instead, the findings can be interpreted as implementation heuristics. Intervention selection should consider delivery feasibility, workforce capacity, supervision structures, cultural acceptability, family context, attendance constraints, safety, and continuity of care. Integrating caregiver support, strengthening facilitator supervision, designing attendance-resilient programmes, and embedding interventions within layered systems of care may enhance implementation. However, these strategies require further empirical validation before they can be treated as established determinants of effectiveness. Clinical PTSD guidance, including broader adult guidance and child/young-person recommendations, supports trauma-focused psychological interventions for clinically significant PTSD symptoms; however, these sources are treated here as contextual clinical guidance rather than direct child-conflict effectiveness evidence [76, 77].

The review also suggests that programme designers should avoid treating delivery platforms as neutral containers for intervention content. Schools, clinics, communities, camps, homes, and digital platforms each create different opportunities and constraints. Platform selection should therefore be based not only on reach, but also on stability, privacy, workforce capacity, referral pathways, and alignment with children’s and families’ needs [14, 15, 17]. Inter-agency MHPSS guidance further supports coordinated, layered care across sectors [78], and KIDNET provides a child- and adolescent-adapted narrative exposure therapy model relevant to trauma-related disorders [79].

### Limitations

The findings should be interpreted in light of several limitations. First, the absence of prospective protocol registration introduces a risk of analytic flexibility. This risk was mitigated through transparent reporting of post hoc methodological amendments, dual screening and extraction, explicit classification rules, and consistent separation of evidence categories.

Second, the inclusion of heterogeneous evidence sources was appropriate for interpretive mapping but limits causal inference. Although effectiveness conclusions were restricted to comparative or controlled intervention studies, variation in outcome measures, intervention content, follow-up periods, delivery settings, and risk-of-bias profiles reduced precision.

Third, the transformation of quantitative findings into directional categories simplified complex effect patterns. This approach was used to support transparent descriptive synthesis, not to substitute for meta-analysis or vote-counting. Non-significant findings were not interpreted as evidence of no effect.

Fourth, JBI appraisal was conducted within study-design categories and should not be interpreted as enabling direct comparison of evidentiary strength across designs. Study-level methodological quality was therefore distinguished from body-of-evidence certainty.

Fifth, contextual or implementation evidence sources were important for interpretation but could not establish causal mechanisms. Associations or interpretive patterns involving caregiver functioning, daily stressors, attendance, supervision, safety, cultural fit, service access, or platform stability should therefore be interpreted as hypothesis-generating.

Sixth, the revised synthesis includes reviews, guidelines, operational guidance, and conceptual sources as contextual or implementation evidence. These sources strengthen ecological and implementation interpretation, but they are not primary intervention studies and were not used to infer intervention effects. For this reason, the review uses the term “evidence sources” where appropriate and separates effectiveness claims from contextual and policy interpretation.

Finally, restriction to English-language publications may have excluded relevant regional evidence, including local evaluations, non-English studies, and humanitarian programme reports. This may have limited representation of locally developed interventions and implementation experience.

## Conclusion

This review reframes psychosocial interventions for children affected by armed conflict as context-dependent systems rather than discrete therapeutic packages. By distinguishing effectiveness evidence from non-comparative intervention evidence and contextual or implementation evidence sources, the review provides a more cautious and methodologically transparent account of the evidence base.

The findings suggest that psychosocial interventions may improve mental health and psychosocial outcomes in some conflict-affected child and adolescent populations, particularly when delivered through structured, supervised, culturally responsive, and stable platforms. Family and caregiving context, delivery continuity, supervision, cultural fit, workforce capacity, and service infrastructure appear important for understanding variation in feasibility and outcomes, but their causal roles remain under-tested.

The contribution of this review lies not in identifying definitive answers, but in offering a structured, theory-informed map of the evidence landscape. Future research should move beyond broad questions of average effectiveness and directly test mechanisms, moderators, implementation conditions, developmental differences, equity, digital access, and long-term outcomes in diverse conflict-affected settings.

## Supplementary Information

Supplementary Material 1.

## Data Availability

No datasets were generated or analysed during the current study.
